# Mining anticoagulant peptides from *Poecilobdella manillensis* by peptidomics analysis

**DOI:** 10.1007/s13659-025-00573-0

**Published:** 2026-02-02

**Authors:** Han-xue Zheng, Xiao-li Deng, Teng-teng Li, Guo-hua Xia, Huan Yang, Jiang-song Peng, Yu-ping Shen

**Affiliations:** Department of Chinese Materia Medica and Pharmacy, School of Pharmacy, Jiangsu University, Zhenjiang, Jiangsu 212013 People’s Republic of China

**Keywords:** *Poecilobdella manillensis*, Peptidomics analysis, Medicinal leech, Anticoagulant peptides, Hirudo

## Abstract

Thrombosis triggers various severe diseases, while antithrombotic drugs carry bleeding risks, making the development of novel natural anticoagulants a subject of widespread attention. *Poecilobdella manillensis*, a prevalent medicinal leech, exhibits remarkable anticoagulant and antithrombotic activities. However, the material basis underlying its anticoagulant effects remains insufficiently investigated. This study aims to mine anticoagulant peptides from *P. manillensis* by peptidomics analysis, elucidate the material basis of its anticoagulant activity, and provide candidate molecules for developing novel natural anticoagulant drugs. Proteins extracted from *P. manillensis* were enzymatically digested and fractionated using DEAE-52 and CN columns. The resulting peptide components were analyzed by UPLC-Q-Orbitrap HRMS, and peptide sequences were matched against proteomic databases using Proteome Discoverer. Anticoagulant peptides were predicted using the BIOPEP-UWM database and PeptideRanker server, followed by in vitro and in vivo activity validation. Results showed that the hydrolysate consisted predominantly of low-molecular-weight peptides. 1533 peptides with Mw < 3000 Da (length < 20 amino acids) were identified from the PM-A2 and PM-A3 fractions, accounting for 40.76% of the total. Four peptides selected through predictive screening demonstrated anticoagulant and antithrombotic activities in vitro. Among them, LE-11 significantly prolonged both APTT and TT (*P* < 0.0001). Furthermore, LE-11 effectively alleviated carrageenan-induced thrombosis in mice, outperforming the heparin control at the mid-concentration (20 mg/kg). In this study, the highly active anticoagulant peptide LE-11 was identified from *P. manillensis* through peptidomic analysis. These findings establish a solid foundation for developing anticoagulant drugs from this source and provide critical scientific support for its clinical application in treating thrombotic diseases.

**Graphical Abstract:**

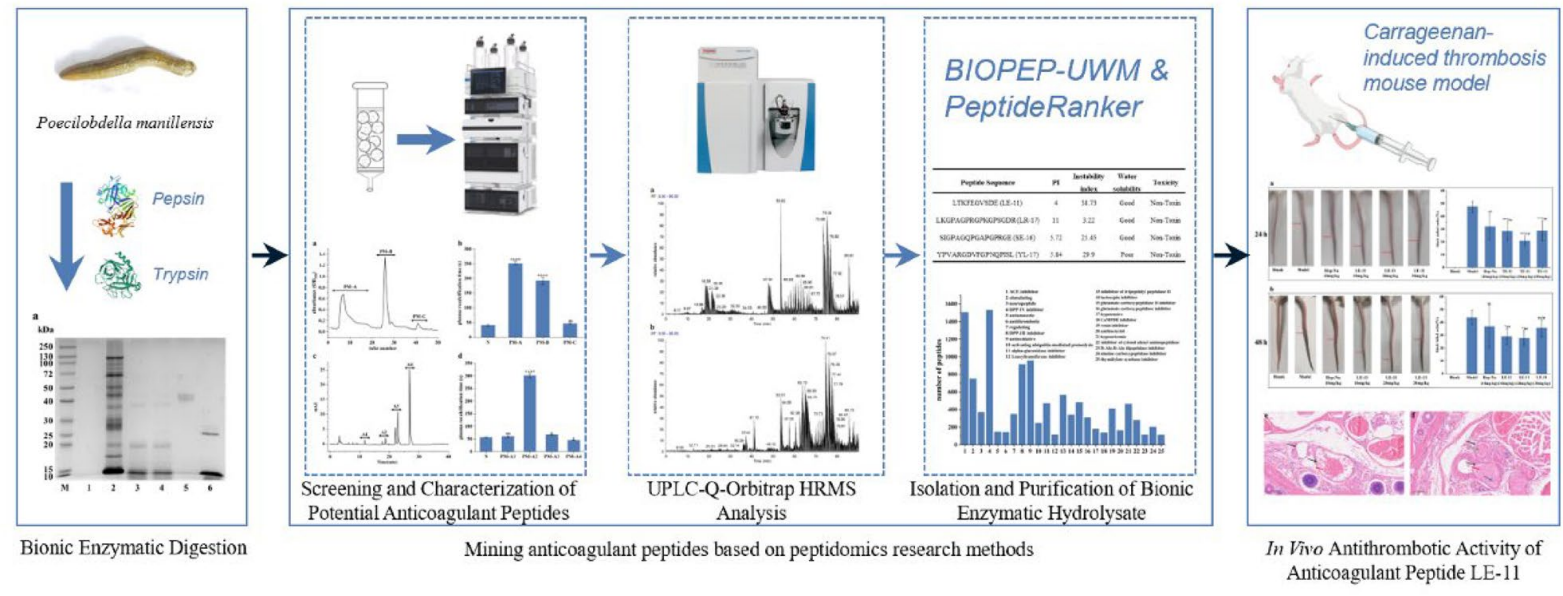

**Supplementary Information:**

The online version contains supplementary material available at 10.1007/s13659-025-00573-0.

## Introduction

A thrombus is a solid mass formed by the abnormal aggregation of blood components within the living cardiovascular system. Thrombosis is a critical factor in arterial thrombotic diseases associated with myocardial infarction and stroke, while venous thromboembolic disorders also contribute significantly to morbidity and mortality [1]. Furthermore, thrombi are the primary cause of most heart attacks and exacerbate other pathological conditions, including various cancers and peripheral vascular diseases [2].

Antithrombotic agents are conventionally classified into three categories: anticoagulants, platelet inhibitors and fibrinolytics. Anticoagulants and platelet inhibitors are primarily used to prevent thrombus formation, whereas fibrinolytics convert plasminogen to plasmin and degrade fibrin, thereby dissolving pre-existing thrombi [3, 4]. Despite their efficacy in alleviating thrombosis, the clinical utility of most antithrombotic drugs is constrained by the risk of bleeding complications, which causes severe consequences [5]. To mitigate these side effects and expand therapeutic options, there is a compelling need to either refine existing agents through purification or develop novel anticoagulants [6].

Medicinal leeches have been utilized in healthcare since antiquity, representing a well-established source of animal-derived medicine with anticoagulant properties [7–9]. Their most renowned salivary product, hirudin, ranks among the most potent natural anticoagulants and was the sole agent available to prevent blood clotting prior to the discovery of heparin. Research on hirudin has subsequently facilitated the development of new anticoagulants, which now serve as cornerstones in the management of thromboembolic diseases [10]. *Poecilobdella manillensis*, a medicinal leech widely employed in Asia [11], also exhibits significant anticoagulant and antithrombotic activities [4, 12]. In clinical applications, *P. manillensis* is typically administered orally as lyophilized powder. The principal active components of *P. manillensis* are proteins and peptides, which are thought to be degraded by gastrointestinal enzymes into bioactive short peptides or amino acids that could constitute the material basis for its anticoagulant and antithrombotic effects [13].

However, the screening of peptide-based molecular entities with specific biological activities from complex mixtures remains challenging. The ongoing advancement of peptidomics has provided novel research paradigms to facilitate the discovery of potential bioactive peptides [14–16]. Peptidomics, first proposed by Schrader et al. [17], plays a pivotal role in the discovery of peptide and peptide-derived drugs. A notable example is the bradykinin-potentiating peptides isolated from the snake *Bothrops jararaca*; which directly inspired the design of captopril, the first angiotensin-converting enzyme (ACE) inhibitor for treating hypertension [18]. Similarly, exendin-4, a long-acting GLP-1 analog derived from lizard venom, was synthesized as exenatide-Byetta and licenzed in 2005 for the treatment of type 2 diabetes. [19]. Furthermore, peptidomics demonstrates broad applications across diverse fields, including immunomodulation, personalized medicine, pharmacognosy, traditional medicine, and food science [20–22].

In this study, proteins extracted from *P. manillensis* were subjected to bionic enzymatic hydrolysis. The resulting hydrolysate was fractionated using DEAE-52 ion-exchange chromatography followed by a cyanogen (CN) column, with fraction collection guided by anticoagulant activity assays to enrich for active components. The anticoagulant fractions were analyzed by UPLC-Q-Orbitrap HRMS. Subsequently, the potential bioactivities of the identified peptides were predicted using the BIOPEP-UWM database and PeptideRanker server. By integrating these predictions with assessments of toxicity and physicochemical properties, four candidate peptides were selected for further validation. Among them, the peptide LE-11 significantly prolonged both the APTT and TT in vitro. Furthermore, LE-11 effectively ameliorated carrageenan-induced thrombosis in mice. Collectively, these results establish a foundation for the development of novel natural anticoagulants derived from *P. manillensis*.

## Results and discussion

### Anticoagulant activity of bionic enzymatic hydrolysate

**Fig. 1:**
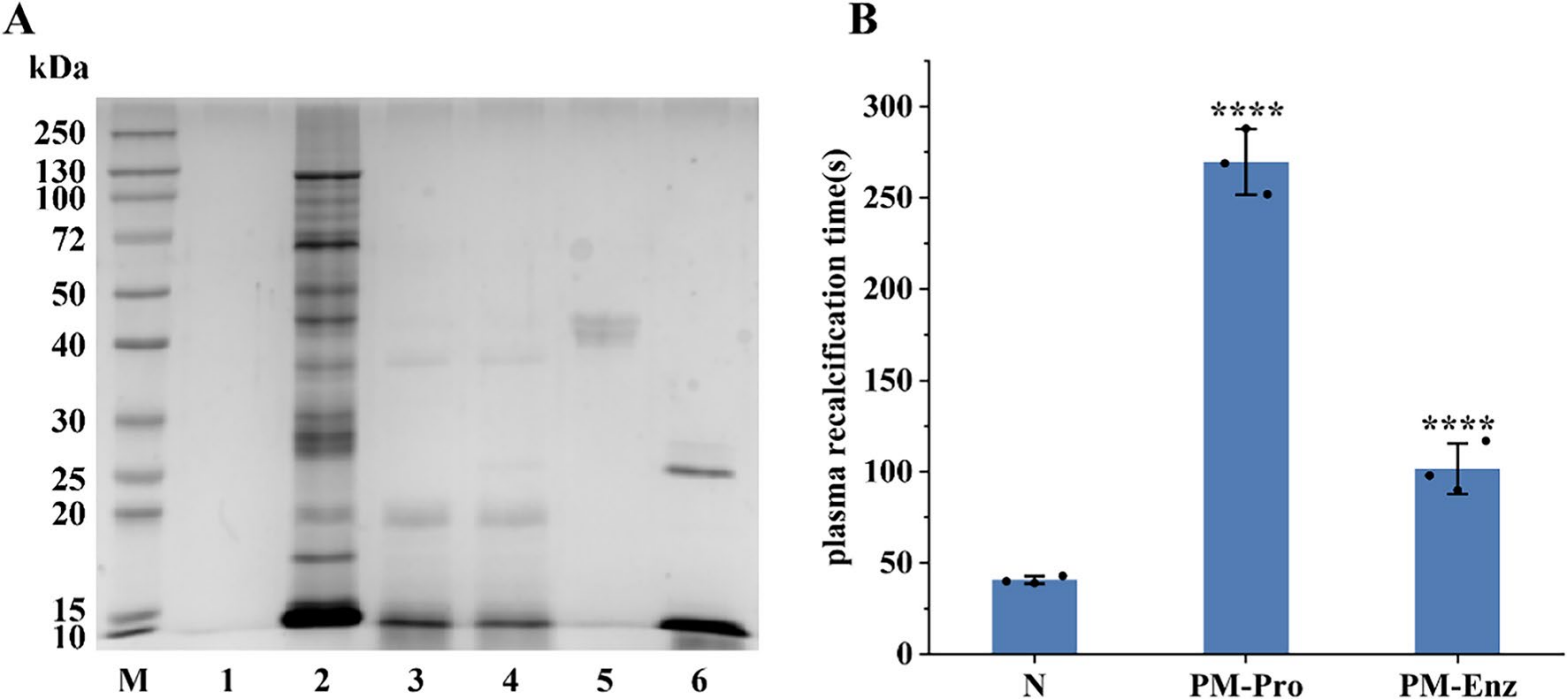
Bionic enzymatic digestion of proteins from *P. manillensis***. A** SDS-PAGE analysis. **B** PRT measurmentof *P. manillensis* proteins before and after biomimetic enzymatic digestion. M: Marker; Lane 1 ~ 6: Blank, PM proteins solution, after pepsin digestion, after trypsin digestion, pepsin, trypsin; PM-Pro: PM proteins; PM-Enz: PM Enzymatic hydrolysate. Values were presented as the mean ± SD (n = 3). ^****^P < 0.0001 for the experimental groups versus the blank control (N)

SDS-PAGE analysis indicated that most protein bands with molecular weights > 10 kDa disappeared following pepsin digestion of the total protein extract from *P. manillensis*. Subsequent digestion with trypsin did not produce significant alterations in the electrophoretic profile, suggesting that the macromolecular proteins had been largely degraded into peptide fragments by the initial pepsin treatment (Fig. 1A). Anticoagulant activity assays showed that plasma recalcification time (PRT) of the hydrolysate was nearly 50% shorter than that of the protein solution prior to bionic enzymatic digestion, indicating a reduction in anticoagulant potency. Nevertheless, the hydrolysate's anticoagulant activity remained statistically extremely significant compared to the blank control (*P* < 0.0001) (Fig. 1B).

### Isolation and purification of bionic enzymatic hydrolysate

**Fig. 2:**
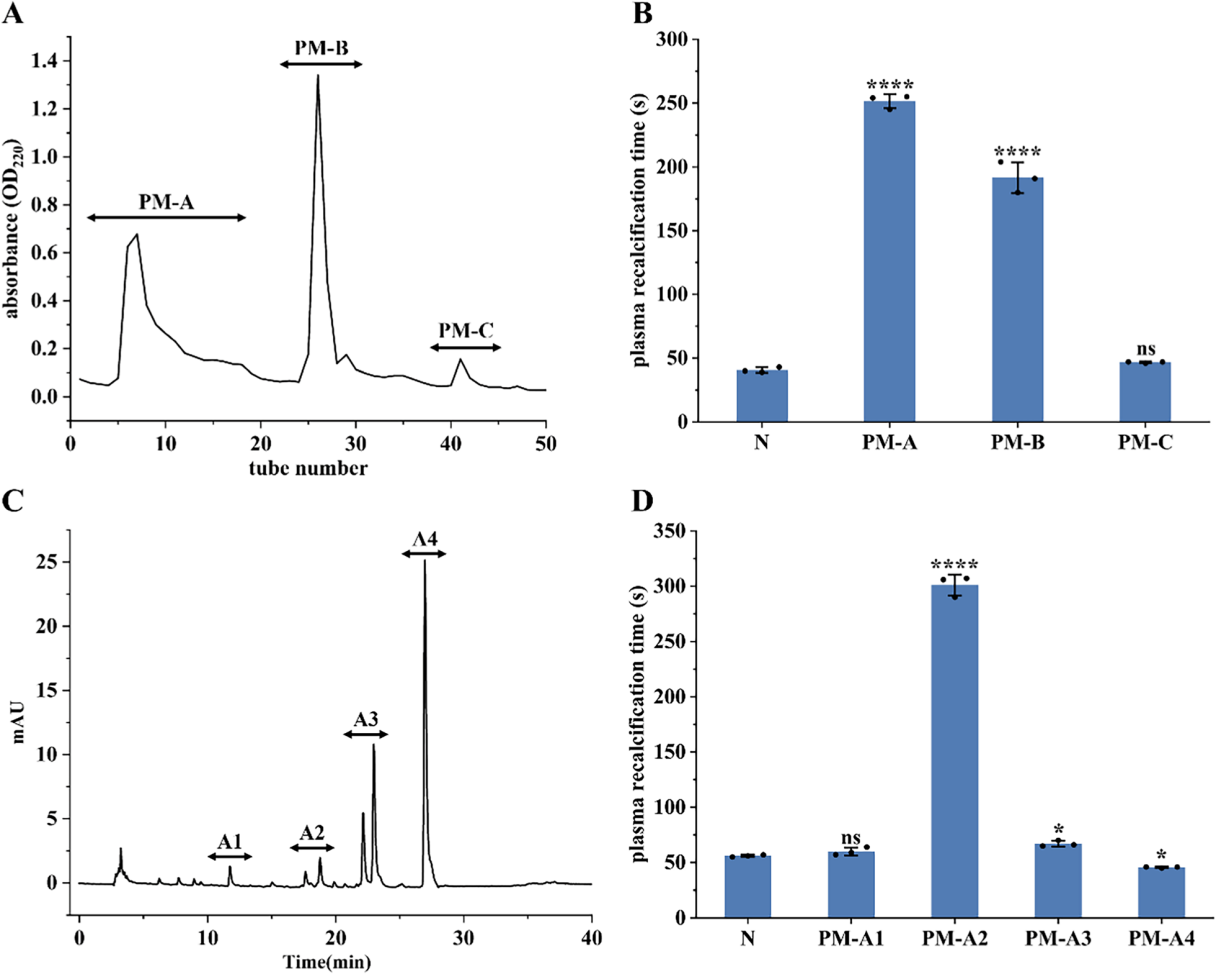
Isolation and anticoagulant activity of bionic enzymatic hydrolysate. **A** DEAE-52 elution profile of *P. manillensis* bionic enzymatic hydrolysate and **B** PRT of three fractions PM-A, PM-B and PM-C. **C** Chromatogram of fraction PM-A and **D** PRT of four subfractions PM-A1, PM-A2, PM-A3 and PM-A4. Values were presented as the mean ± SD (n = 3). ****P < 0.0001 and *P < 0.05 for the experimental groups versus the blank control (N). ns, no significance

The enzymatic hydrolysate of *P. manillensis* was fractionated using a DEAE-52 ion-exchange column, yielding three primary fractions designated as PM-A, PM-B and PM-C (Fig. 2A). Each fraction was desalted via a C18 solid-phase extraction column, lyophilized, and reconstituted for PRT assay (Fig. 2B). Both PM-A and PM-B significantly prolonged the PRT compared to the blank control (*P* < 0.0001), indicating potent anticoagulant activity, with PM-A exhibiting the strongest effect. In contrast, PM-C showed no significant difference from the control (*P* > 0.05). Given its superior activity, fraction PM-A was selected for further separation on a CN column, resulting in four subfractions (PM-A1, PM-A2, PM-A3, PM-A4) (Fig. 2C), which were collected and lyophilized. PRT analysis of these subfractions (Fig. 2D) revealed distinct activities: PM-A1 showed no significant difference from the blank control; PM-A2 exhibited extremely significant anticoagulant activity (*P* < 0.0001); PM-A3 also showed significant activity (*P* < 0.05); whereas PM-A4 demonstrated procoagulant activity. Based on these results, the two anticoagulant-active fractions, PM-A2 and PM-A3, were selected for subsequent peptidomics analysis by mass spectrometry, combined with bioinformatics screening, to identify potential anticoagulant peptides from *P. manillensis*.

### UPLC-Q-Orbitrap HRMS analysis

**Fig. 3:**
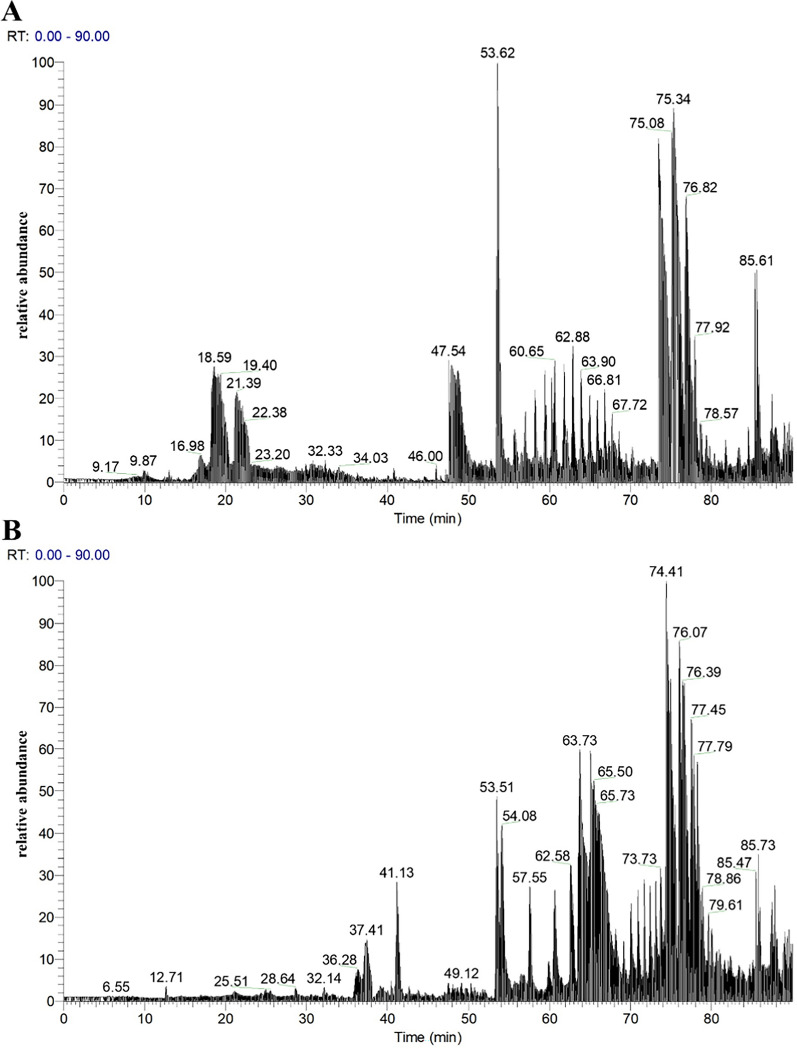
Total ion chromatograms of fractions PM-A2 (**A**) and PM-A3 (**B**)

**Fig. 4:**
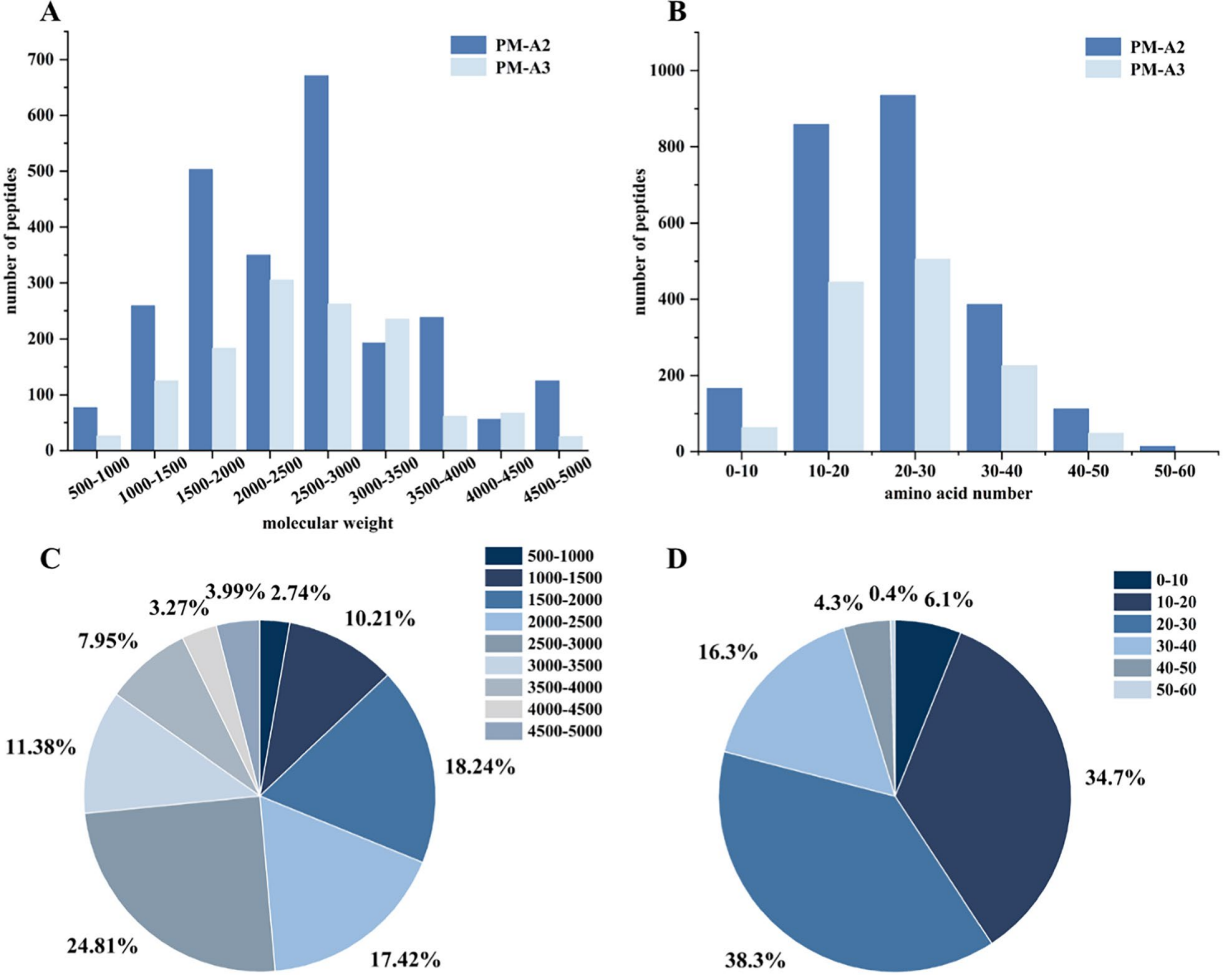
The relative molecular weight and amino acid number distribution of peptides contained in subfractions PM-A2 and PM-A3

The total ion chromatograms of fractions PM-A2 and PM-A3, acquired in positive ion mode, were shown in Fig. 3. Raw MS data were processed using Proteome Discoverer 2.4 for peptide identification. Duplicate peptide sequences were removed, retaining the longer sequence when identical peptides were detected. This analysis identified 2472 and 1289 peptide sequences in fractions PM-A2 and PM-A3, respectively. It can be seen from Fig. 4. that peptides in both fractions were predominantly distributed within three molecular mass ranges: 1500–2000 Da, 2000–2500 Da, and 2500–3000 Da, collectively accounting for 73.41% of the total. Correspondingly, peptide lengths were primarily concentrated in the 10–20 and 20–30 amino acid ranges, constituting 72.96% of all identified sequences.

Notably, numerous studies have reported that anticoagulant and antithrombotic peptides typically belong to the oligopeptide category, generally comprising fewer than 20 amino acids and possessing molecular weights under 3000 Da [23–27]. Statistical analysis showed that fractions PM-A2 and PM-A3 contained 1533 peptides meeting these criteria, which represented 40.76% of all identified peptides. This substantial proportion underscored the significant abundance of potential anticoagulant peptides in these fractions.

### Screening and characterization of potential anticoagulant peptides

**Fig. 5:**
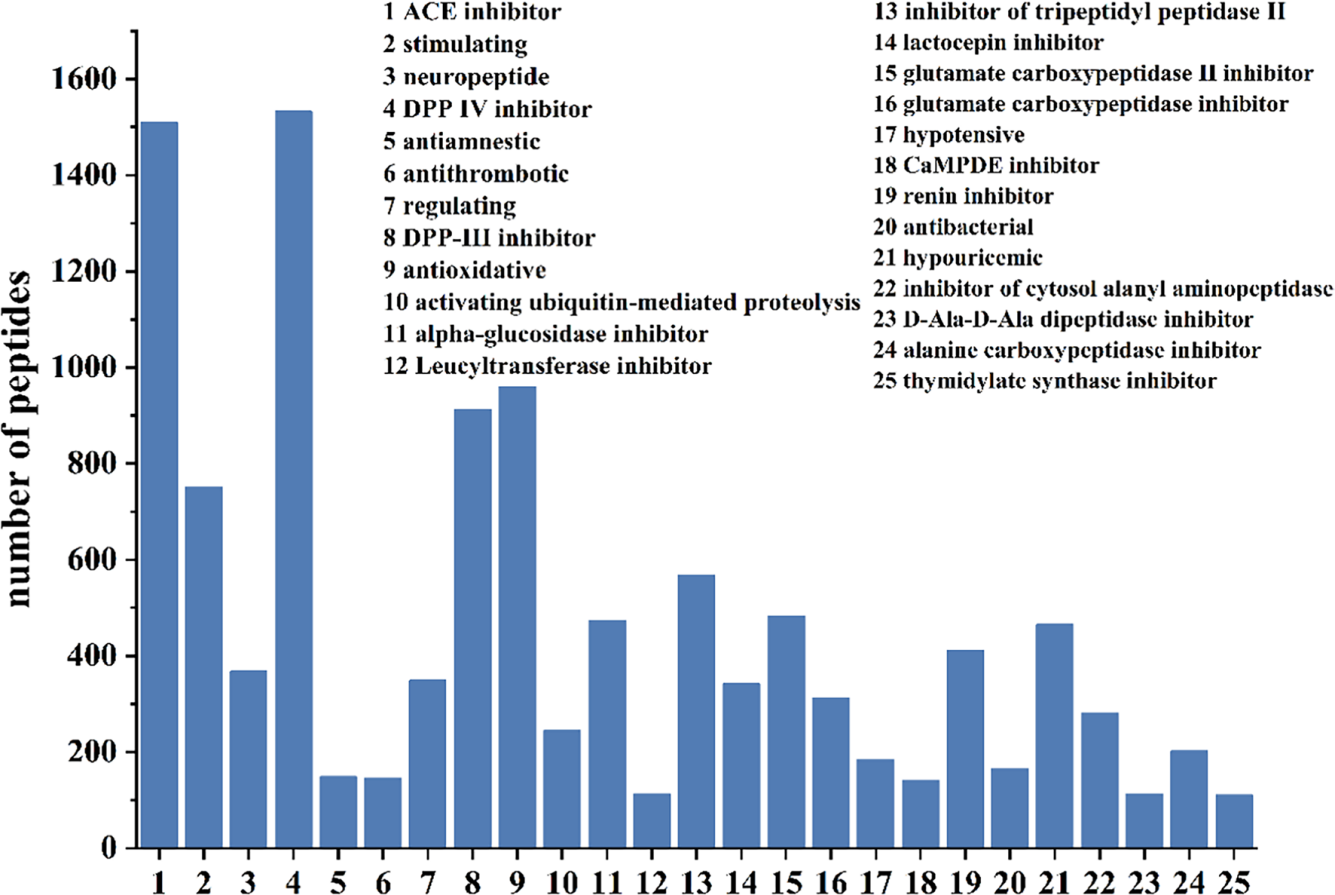
Bioactivity prediction of peptides in subfractions PM-A2 and PM-A3

The potential bioactivities of the aforementioned 1,533 peptides were predicted using the BIOPEP-UWM database (Fig. 5). The results indicated that nearly all peptides exhibited potential dipeptidyl peptidase IV (DPP IV) and angiotensin-converting enzyme (ACE) inhibitory activities. In contrast, 145 peptides were predicted to possess potential antithrombotic activity. Given that peptide motifs such as DEE, KRDS, PPK, GPGG, GPRGP and RGD have been extensively documented to exert antithrombotic effects through diverse mechanisms [28–32], a subsequent screening of the 145 candidate peptides for these sequences was performed. This process identified 26 peptides containing the aforementioned motifs for further analysis.

The bioactivity potential of these 26 peptides was subsequently predicted using the PeptideRanker server **(**Table 1**)**. Further screening was performed by integrating BIOPEP parameter A (frequency of bioactive peptide occurrence in proteins) and B (probability or intensity of a peptide exhibiting specific bioactivity). This multi-criteria screening ultimately identified four candidate peptides: SIGPAGQPGAPGPRGE (SE-16), LKGPAGPRGPKGPSGDR (LR-17), LTKFEGVSDEE (LE-11), and YPVARGDVFGPNQPISL (YL-17) (bold and bolditalics in Table 1; MS^2^ shown in Fig. S1). Thus, a total of four peptides with promising anticoagulant and antithrombotic potential were successfully screened from the *P. manillensis* enzymatic hydrolysate.

**Table 1:** Information of potential anticoagulant and antithrombotic peptides

No.	Peptide sequence	Length	Activity	Parameter B	Parameter A	BIOPEP-ID
1	***SIGPAGQPGAPGPRGE***	**16**	**0.7134**	–	**0.4375**	**GPRG (3040)** **GPR (3047)** **GP (3283)** **PGP (3284)** **PG (3285)**
2	***LKGPAGPRGPKGPSGDR*** ***N-Term (Acetyl)***	**17**	**0.6654**	–	**0.4118**	**GPRG (3040)** **GPRGP (3041)** **GPR (3047)** **GP (3283)**
3	RGSVSRNQNLQGPPKP	16	0.1580	–	0.125	PPK (8168); GP (3283)
4	RGAAVSAGPRKAVIEE	16	0.1482	–	0.125	GPR (3047)
5	***YPVARGDVFGPNQPISL***	**17**	**0.4414**	–	**0.1176**	**RGD (9660)** **GP (3283)**
6	EISFGPRVLTNFASMITPA M15 (Oxidation)	19	0.4891	–	0.1053	GPR (3047) GP (3283)
7	FEKWKNPPKANF	12	0.7472	–	0.0833	PPK (8168)
8	FLFPPKPKDTLM	12	0.8934	–	0.0833	PPK (8168)
9	LNTSVVMPPKDFLEL M7 (Oxidation)	15	0.2797	–	0.0667	PPK (8168)
10	PIFGKNSKRDSPLKNVF N-Term (Acetyl)	17	0.6631	–	0.0588	KRDS (3290)
11	QPIHQPTDPSITPVTNPPK	19	0.2974	–	0.0526	PPK (8168)
12	KAPPKVETDTPKKMEPAPKP N-Term (Acetyl); M14 (Oxidation)	20	0.3018	–	0.05	PPK (8168)
13	***LTKFEGVSDEE***	**11**	**0.0503**	**0.0002**	**0.0909**	**DEE (3354)**
14	HDDDDDEEDDDN	12	0.0640	0.00018	0.0833	DEE (3354)
15	EGEEEDEESEDE	12	0.0315	0.00018	0.0833	DEE (3354)
16	SDEEDVDDSEED N-Term (Acetyl)	12	0.0497	0.00018	0.0833	DEE (3354)
17	EDAESEDEEEED	12	0.0320	0.00018	0.0833	DEE (3354)
18	HDDDDDEEEDDDD	13	0.0397	0.00017	0.0769	DEE (3354)
19	HDDDDDDDEEDDE	13	0.0559	0.00017	0.0769	DEE (3354)
20	GAVEEFSDDEEKFQ	14	0.1337	0.00016	0.0714	DEE (3354)
21	DEELTHVEEQLSGL	14	0.1107	0.00015	0.0714	DEE (3354)
22	HDDDDDDGDDEEDDN	15	0.0566	0.00014	0.0667	DEE (3354)
23	EPEHEEDEEGDEQEA	15	0.0484	0.00014	0.0667	DEE (3354)
24	VVIVTLNGDDDEEDDD N-Term (Acetyl)	16	0.0337	0.00014	0.0625	DEE (3354)
25	KHLDDEELIKIGVTIQMH	18	0.3404	0.00012	0.0556	DEE (3354)
26	HDDDHDDDHDEETEKPPSE	19	0.0670	0.00011	0.0526	DEE (3354)

The potential toxicity of the four candidate peptides (LE-11, LR-17, SE-16, YL-17) was assessed using the ToxinPred server, while physicochemical properties including theoretical isoelectric point, hydrophilicity/hydrophobicity, and stability were predicted with the Expasy-ProtParam tool (Table 2). The predictions indicated that none of the peptides were toxic. Moreover, all peptides except YL-17 were predicted to have good water solubility. The instability indices for all four peptides were below 40, suggesting favorable stability profiles.

**Table 2:** Prediction of physicochemical properties and toxicity of potential anticoagulant and antithrombotic peptides

Peptide sequence	PI	Instability index	Water solubility	Toxicity
LTKFEGVSDEE (LE-11)	4	38.73	Good	Non-toxin
LKGPAGPRGPKGPSGDR (LR-17)	11	3.22	Good	Non-toxin
SIGPAGQPGAPGPRGE (SE-16)	5.72	25.45	Good	Non-toxin
YPVARGDVFGPNQPISL (YL-17)	5.84	29.9	Poor	Non-toxin

### In vitro activity of anticoagulant peptides

**Fig. 6:**
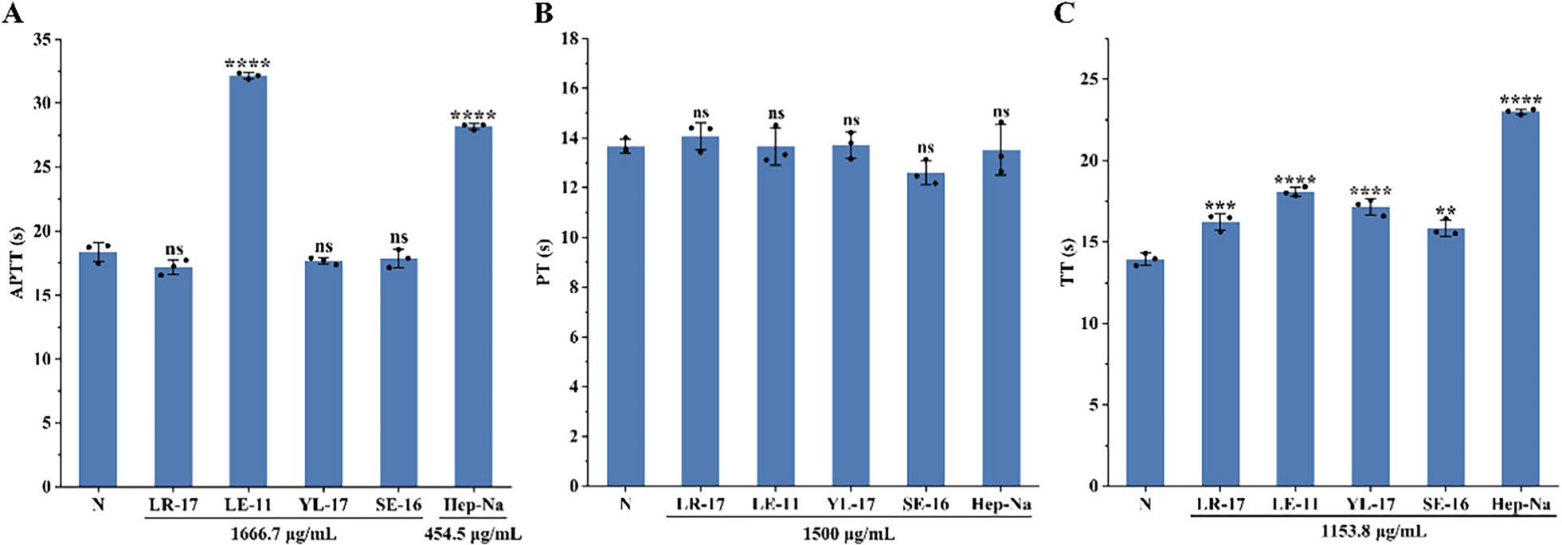
Assay of APTT (**A**), PT (**B**) and TT (**C**) of synthetic peptides. Values were presented as the mean ± SD (n = 3). ^**^P < 0.01, ^***^P < 0.001 and ^****^P < 0.0001 for the experimental and positive groups versus the blank control (N). ns, no significance

The anticoagulant activities of peptides LE-11, LR-17, SE-16, and YL-17 were evaluated by measuring their effects on activated partial thromboplastin time (APTT), prothrombin time (PT), and thrombin time (TT). As shown in Fig. 6, LE-11 significantly prolonged APTT compared to the blank control (*P* < 0.0001), whereas LR-17, YL-17, and SE-16 had no significant effect. All four peptides significantly prolonged TT, with LE-11 again showing the most potent effect (*P* < 0.0001). In contrast, none of the peptides induced a significant change in PT. These findings indicated that LE-11 possesses notable anticoagulant activity in vitro, likely mediated through the intrinsic coagulation pathway and the conversion of fibrinogen to fibrin.

**Fig. 7:**
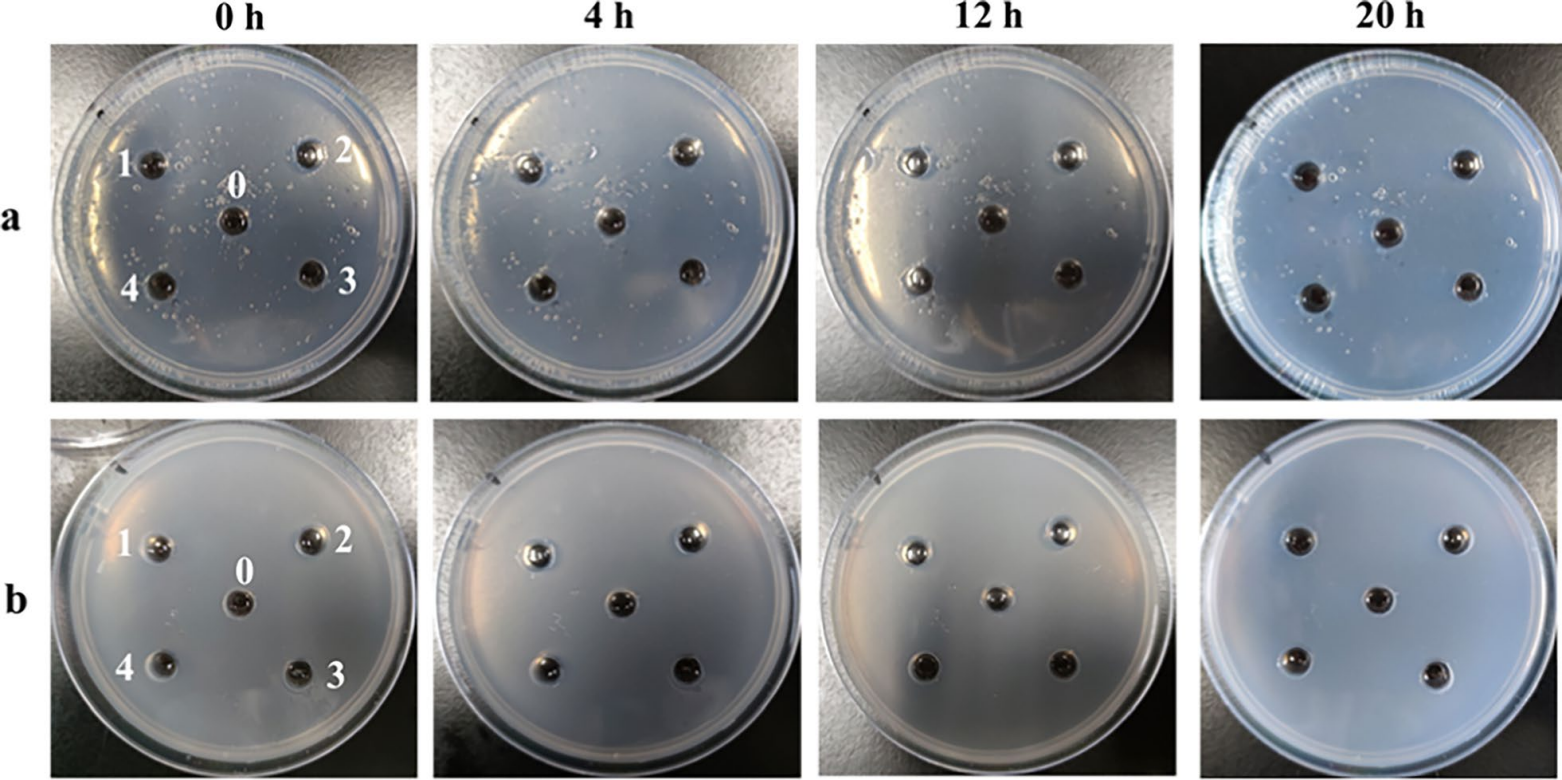
Assay of fibrinolytic activity of synthetic peptides. **a** Fibrinogen plate; **b** fibrin plate; 0: blank; 1: LR-17; 2: LE-11; 3: YL-17; 4: SE-16

To investigate direct fibrinolytic effects of the peptides, fibrinogen and fibrin plate assays were conducted. As shown in Fig. 7, none of the four peptides produced a discernible dissolution zone on either plate type, indicating a lack of direct interaction with fibrinogen or fibrin. Consequently, the antithrombotic activity of these peptides did not involve direct fibrinolytic activity.

### In vivo antithrombotic activity of anticoagulant peptide LE-11

**Fig. 8:**
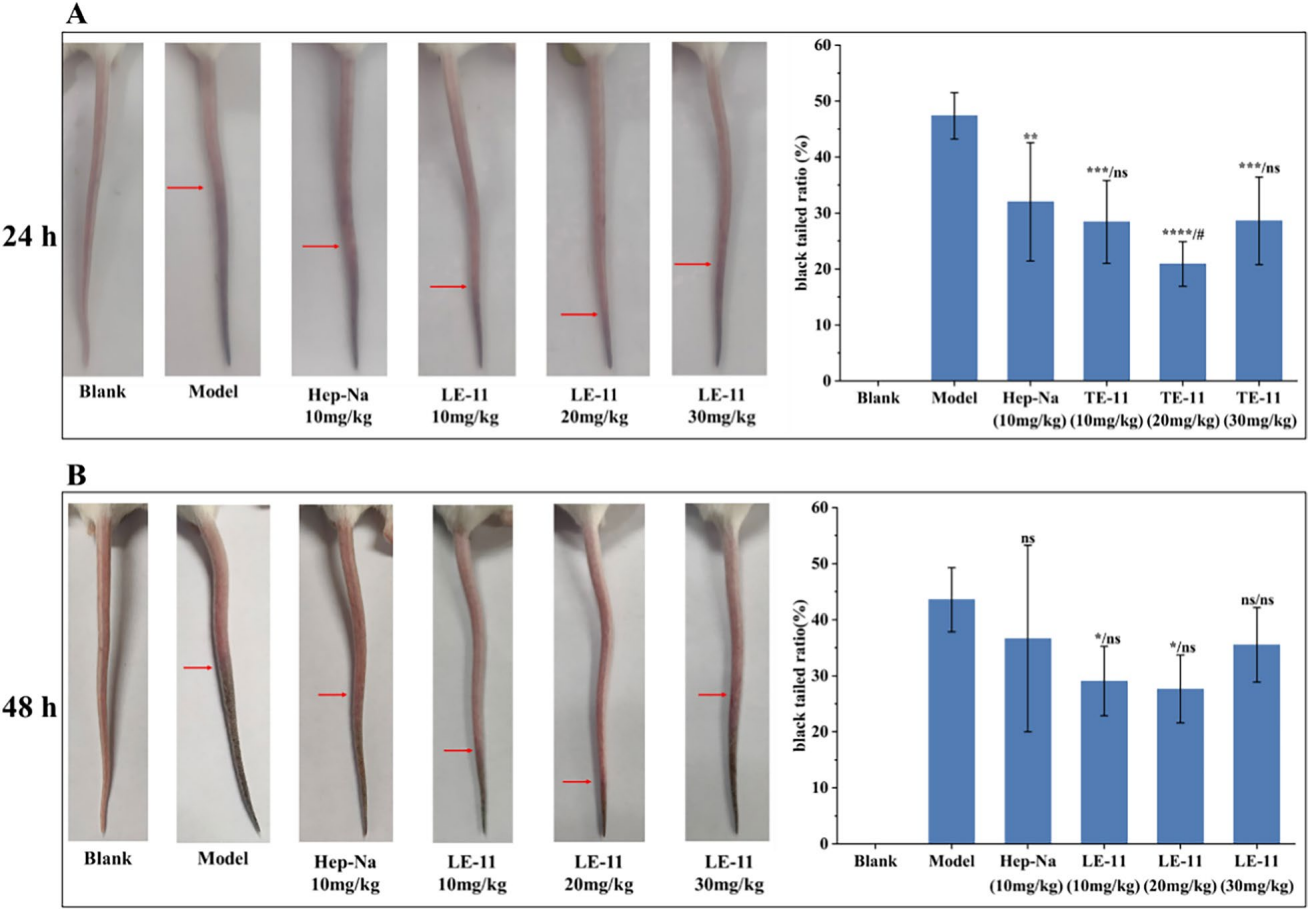
The effect of LE-11 on tail blackening length and black tailed ratio in mice at 24 h (**A**) and 48 h (**B**) after carrageenan modeling. Values were presented as the mean ± SD (n = 6). ^*^P < 0.05, ^**^P < 0.01, ^***^P < 0.001 and ^****^P < 0.0001 for the experimental and heparin control groups versus the model. ^#^*P* < 0.05 for the experimental groups versus the heparin control group. ns, no significance

The antithrombotic efficacy of LE-11 was evaluated using a carrageenan-induced thrombosis model in mice. As shown in Fig. 8, tail blackening was observed after 24 and 48 h of modeling, indicating successful thrombus formation; the extent of blackening intensified over time. Compared to the model group, both the heparin control and LE-11 treatment groups exhibited varying degrees of improvement in tail blackening. At the 24 h time point, the medium-dose LE-11 group (20 mg/kg) showed a significantly lower blackened tail ratio than the heparin group (*P* < 0.05), suggesting superior antithrombotic efficacy at this stage. By 48 h, the low- (10 mg/kg) and medium-dose LE-11 groups still maintained a significantly reduced blackened tail ratio relative to the model group (*P* < 0.05), with no statistical difference from the heparin control.

**Fig. 9:**
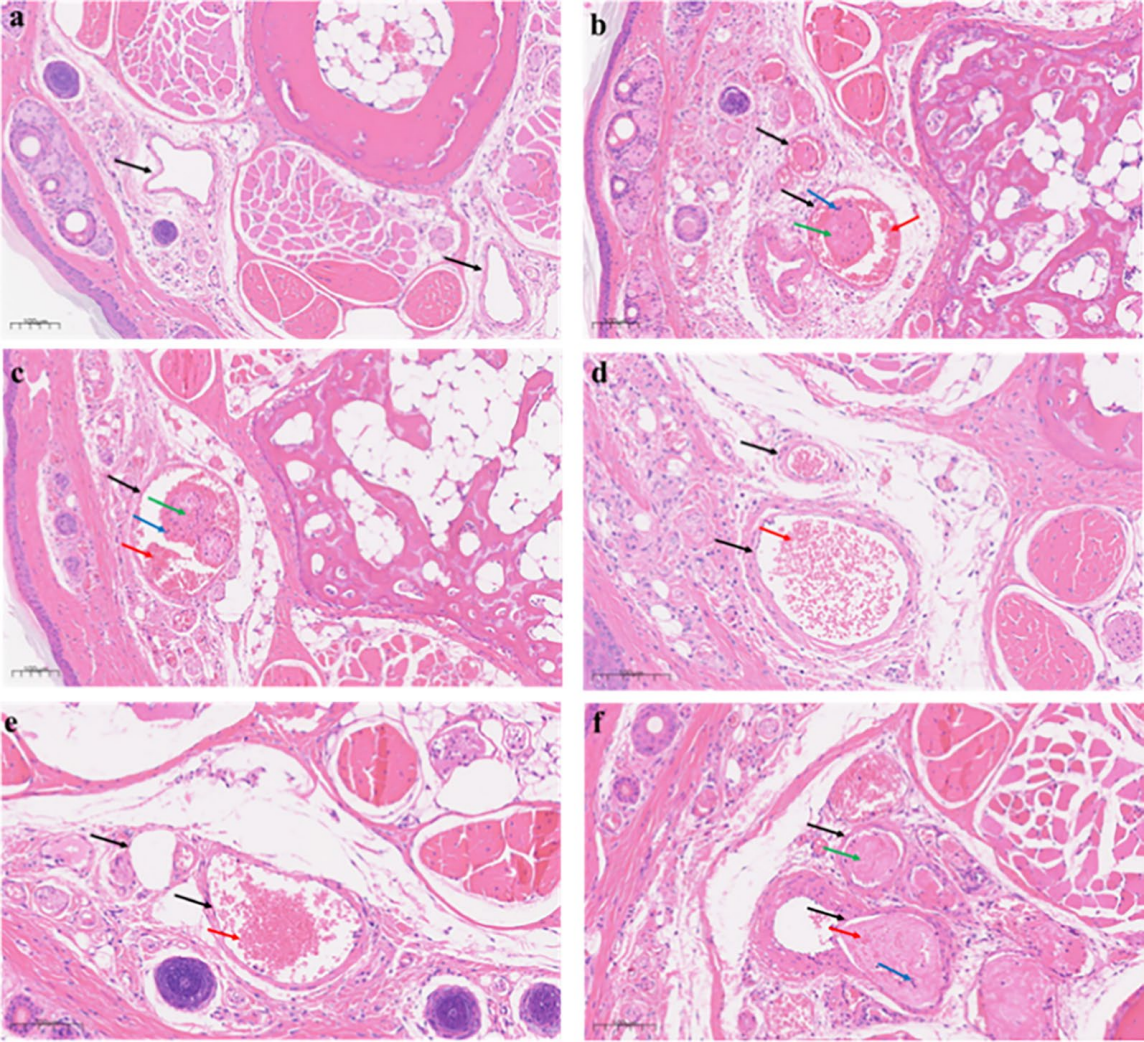
Effect of LE-11 on the histomorphology of mouse tail vein thrombi. **a** Blank, **b** Model, **c** Hep-Na, **d** low-dose group, **e** medium-dose group, **f** high-dose group

To further corroborate the inhibitory effect of LE-11 on thrombosis, transverse tail tissue sections (approximately 3 cm from the tip) were prepared and subjected to hematoxylin and eosin (H&E) staining. As shown in Fig. 9, the blank control group exhibited blood vessels with clear architecture and patent lumens, with no thrombi present. In contrast, the model group displayed extensive inflammatory cell infiltration and occlusive thrombi that nearly filled the vascular lumina, characterized by densely cross-linked fibrin networks. Compared to the model group, both the heparin control and all LE-11 treatment groups showed substantial mitigation of thrombotic pathology. Notably, in the low- and medium-dose LE-11 groups, thrombus formation was markedly suppressed, with only erythrocyte aggregates observed in the lumina and an absence of fibrin scaffold formation, underscoring a pronounced antithrombotic effect.

## Conclusions

This study isolated peptides from the enzymatic hydrolysate of *P. manillensis* proteins using an anticoagulant bioactivity-guided approach. The hydrolysate was initially fractionated by DEAE-52 ion-exchange chromatography, yielding three fractions (PM-A, PM-B, and PM-C), of which PM-A exhibited the most potent anticoagulant activity. Further purification of PM-A via CN column chromatography produced two bioactive subfractions, PM-A2 and PM-A3. Analysis of these subfractions by UPLC-Q-Orbitrap HRMS identified a total of 1,533 peptides with a molecular weight below 3,000 Da and fewer than 20 amino acids, accounting for 40.76% of all detected peptides. Bioactivity prediction using the BIOPEP-UWM database and PeptideRanker server led to the selection of four candidate peptides (LE-11, LR-17, SE-16, and YL-17) with potential anticoagulant and antithrombotic activities. In vitro assays confirmed that LE-11 (LTKFEGVSDEE) significantly prolonged both APTT and TT, suggesting an anticoagulant mechanism involving the intrinsic coagulation pathway and the conversion of fibrinogen to fibrin. In vivo evaluation further demonstrated that LE-11 effectively ameliorated carrageenan-induced thrombosis in mice, with medium-dose administration (20 mg/kg) exhibiting superior efficacy to the heparin control. In summary, this study successfully identified LE-11 as a potent anticoagulant peptide from *P. manillensis* through a peptidomics-based strategy, laying a scientific foundation for developing novel antithrombotic therapeutics. To promote the development of LE-11, toxicological screening will be subsequently performed, including hemolysis assays and basic cell viability screens for de-risking toxicity.

## Materials and methods

### Materials

DEAE Cellulose (DE-52) and C18 solid-phase extraction columns were supplied by Waters Corporation. A ZORBAX 300SB-CN column (4.6 mm I.D. × 250 mm L., 5 μm) was sourced from Agilent Technologies (USA). Assay kits for APTT, PT, and TT were procured from ZCIBIO Technology Co., Ltd (China). Peptides LE-11, LR-17, SE-16, and YL-17 were synthesized by Nanjing Synpeptide Co., Ltd. using solid-phase synthesis, with purities exceeding 95% as confirmed by high-performance liquid chromatography (HPLC) (Fig. S2). The corresponding HPLC chromatograms and MS/MS spectra were provided in Fig. S3 and S4, respectively.

### Animals

Live leeches of *P. manillensis* were acquired from Guangdong Province, China, and identified by COI barcoding. Following a 48-h fasting period, the leeches were rinsed with sterile physiological saline, lyophilized, and ground into powder. Proteins were subsequently extracted from the powdered material using ultrasonic-assisted extraction. The resulting extract was lyophilized to obtain *P. manillensis* protein powder, which was then stored at -80°C for further use.

Conventional-grade New Zealand rabbits were sourced from the Laboratory Animal Research Center of Jiangsu University (License No. SYXK (Su) 2023–0080). Male BALB/c mice (7 weeks old, SPF grade) were obtained from the same center and maintained under SPF conditions (License No. SCXK (Su) 2023–0017). All experimental procedures involving animals were reviewed and approved by the Institutional Animal Care and Use Committee of Jiangsu University (Approval No. UJS-IACUC-2025011602).

### Measurement of plasma recalcification time

Platelet-poor plasma (PPP) was prepared by collecting blood from the marginal ear vein of healthy New Zealand rabbits and anticoagulating it with 0.15 *M* sodium citrate at a 1:9 (*v/v*) ratio. After gentle mixing, the blood was centrifuged at 3,000 rpm for 10 min, and the supernatant was collected as PPP. For the PRT assay, 100 µL of PPP was combined with 100 µL of the test sample solution in a 96-well plate, using deionized water as the blank control. The plate was incubated at 37 °C for 1 min, after which 100 µL of 0.025 *M* CaCl_2_ solution was added to each well to initiate coagulation. The mixture was continuously stirred, and the time from CaCl_2_ addition to the appearance of fibrin threads or clot formation was recorded as the PRT. This parameter was used to evaluate the anticoagulant activity of the tested samples.

### Bionic enzymatic digestion

The lyophilized protein powder was dissolved in deionized water to yield a 2 mg/mL solution. This solution was adjusted to pH 2.0 using dilute hydrochloric acid and pre-incubated at 37 °C for 5 min. Pepsin was then added at an enzyme-to-substrate ratio of 7% (*w/w*), and the digestion was allowed to proceed at 37 °C for 4 h. Subsequently, the mixture was brought to pH 8.0 by dropwise addition of NaOH solution and incubated again at 37 °C for 5 min. Trypsin was introduced at the same enzyme-to-substrate ratio (7%, *w/w*), and digestion continued for 2 h at 37 °C. To terminate the reaction, the mixture was heated at 85 °C for 15 min to inactivate the enzymes. The resulting hydrolysate was adjusted to pH 7.0 and centrifuged at 8000 × g for 10 min. The supernatant was collected and lyophilized, yielding the final peptide preparation from *P. manillensis*. The efficiency of the bionic enzymatic digestion was assessed by 12% SDS-PAGE with Coomassie Blue staining.

### Purification of enzymatic peptides

The lyophilized bionic enzymatic hydrolysate was dissolved in PBS (pH 6.8) to obtain a 10 mg/mL solution. After centrifugation at 8000 rpm for 10 min, the supernatant was loaded onto a DEAE-52 anion-exchange column (1.6 cm I.D. × 50 cm L.; particle size 40–160 μm) pre-equilibrated with the same PBS buffer. The column was then eluted stepwise with a 0.3–1.0 *M* NaCl gradient. Fractions (8 mL/tube) were collected automatically, and their absorbance at 220 nm was measured to generate the elution profile. Fractions exhibiting anticoagulant activity were pooled, desalted using a C18 solid-phase extraction column, and lyophilized. The anticoagulant activity of each fraction was assessed in vitro.

The most active fractions from the initial separation were selected for further purification on a ZORBAX 300SB-CN column (4.6 mm I.D. × 250 mm L., 5 μm) using an HPLC system. The mobile phase consisted of 0.1% trifluoroacetic acid in water (solvent A) and 0.1% trifluoroacetic acid in acetonitrile (solvent B). Elution was performed at a flow rate of 1 mL/min with the following gradient: 0 min, 98% A: 2% B → 65 min, 20% A: 80% B → 68 min, 98% A: 2% B → 70 min, 98% A: 2% B. The eluate was monitored at 220 and 280 nm with a diode array detector. Target peaks were collected, lyophilized, and subjected to anticoagulant activity assays.

### Identification and analysis by UPLC-Q-Orbitrap HRMS

Fractions PM-A2 and PM-A3 were desalted via C18 solid-phase extraction, lyophilized, and reconstituted in deionized water for analysis using ultra-performance liquid chromatography coupled with quadrupole Orbitrap high-resolution mass spectrometry (UPLC-Q-Orbitrap HRMS).

Chromatographic separation was performed on a PepMap C18 Nano analytical column (50 μm I.D. × 150 mm L., 2 μm) maintained at 55 °C. The mobile phase consisted of 0.1% formic acid in water (solvent A) and 0.1% formic acid in acetonitrile (solvent B). Elution was carried out at a flow rate of 0.35 μL/min with the following gradient: 0 min, 99% A: 1% B → 3 min, 94% A: 6% B → 6 min, 93% A: 7% B → 60 min, 70% A: 30% B → 75 min, 60% A: 40% B → 85 min, 10% A: 90% B → 90 min, 10% A: 90% B. The injection volume was 1 μL. Mass spectrometric detection was conducted with an electrospray ionization (ESI) source in positive ion mode, using a spray voltage of 2.0 kV and a capillary temperature of 320 °C. Additional MS parameters were provided in Table S1.

The raw MS data were processed with Proteome Discoverer 2.4 software and searched against custom protein sequence databases (SRR5135713_WP, SRR7411950_HN, SRR20718731_PM, and hirudinea_0407). An overview of the constructed proteome databases was shown in Fig. S5, and the detailed search parameters were listed in Table S2.

### Screening and property prediction of anticoagulant peptides

The potential bioactivities of peptides derived from enzymatic digestion were predicted using the BIOPEP-UWM database and the PeptideRanker server to identify candidates with anticoagulant properties. Peptides selected based on these predictions were subsequently characterized for toxicity using the ToxinPred database, while physicochemical properties, including theoretical isoelectric point (pI), hydrophobicity and stability, were predicted with the Expasy-ProtParam tool.

### Activated partial thromboplastin time, prothrombin time, and thrombin time assays

For the APTT assay, 50 µL of platelet-poor plasma (PPP) was mixed with 50 µL of peptide solution and 50 µL of APTT reagent. The mixture was incubated at 37 °C for 5 min, after which 50 µL of pre-warmed 25 *mM* CaCl_2_ solution (37 °C) was added to initiate coagulation. In the PT assay, 70 µL of PPP was combined with 30 µL of peptide solution and incubated at 37 °C for 3 min, followed by the addition of 140 µL of pre-warmed PT reagent. For the TT assay, 100 µL of PPP was mixed with 30 µL of peptide solution, incubated at 37 °C for 5 min, and then 100 µL of TT reagent was added. In all assays, timing commenced immediately upon the addition of the final reagent under continuous stirring and was stopped when fibrin threads formed. Deionized water was used as the blank control in all experiments.

### Fibrinolytic activity determination in vitro

Fibrinolytic activity was assessed using fibrinogen and fibrin plates. 100 µL solution of each peptide (2.5 mg/mL) was applied to individual wells on both plate types, with deionized water serving as the blank control. All treatments were performed in triplicate. The plates were then incubated at 37 °C for 20 h. Following incubation, fibrinolytic activity was quantified by measuring the maximum (*D*_*max*_) and minimum (*D*_*min*_) diameters of the dissolution zones. The dissolution area (*S*) was calculated using Eq. 1:

1 $$S\left({mm}^{2}\right)={\left[\left({D}_{max}+{D}_{min}\right)/4\right]}^{2}\times \pi$$

### In vivo antithrombotic activity of anticoagulant peptide LE-11

After a one-week acclimatization period, male BALB/c mice were randomly allocated to six experimental groups (n = 6): a blank control group (normal saline, i.p.), a heparin control group (10 mg/kg heparin sodium, i.p.), a model group (normal saline, i.p.), and three LE-11 treatment groups receiving low (10 mg/kg, i.p.), medium (20 mg/kg, i.p.), and high (30 mg/kg, i.p.) doses.

Mice in the peptide treatment groups received daily intraperitoneal injections of LE-11 for seven consecutive days, whereas those in the blank and model groups were administered an equivalent volume of normal saline. Mice in the heparin control group received a single intraperitoneal injection of heparin sodium on day 7. One hour after the final administration, thrombosis was induced in all groups except the blank control by intraperitoneal injection of 50 mg/kg 0.5% carrageenan solution; the blank control group received an equal volume of normal saline.

Tail blackening length was measured at 24 and 48 h after carrageenan injection, and the blackened tail ratio was calculated for each group. At 48 h, mice were euthanized, and a 3 cm segment of the distal tail was collected and fixed in 4% paraformaldehyde. Following fixation, tissue samples were dehydrated, decalcified, embedded in paraffin, sectioned, and stained with hematoxylin and eosin (H&E). Pathological alterations in the tail tissues were then examined microscopically for intergroup comparison.

### Statistical analysis

For statistical analysis, the data acquired from independent experiments were presented as mean ± SD. One-way ANOVA was used for multiple comparisons by the IBM SPSS Statistics software package. The difference was considered statistically significant if the *P* value < 0.05.

## Supplementary Information

Additional file1 (DOCX 1163 kb)

## Data Availability

The datasets generated and/or analysed during the current study are available from the corresponding author on reasonable request.

## Abbreviations

PM: *Poecilobdella manillensis*;
PRT: Plasma recalcification time;
APTT: Activated partial thromboplastin time;
PT: Prothrombin time;
TT: Thrombin time;
PPP: Platelet-poor plasma;
HPLC: High performance liquid chromatography;
UPLC-Q-Orbitrap HRMS: Ultra-performance liquid chromatography-quadrupole-orbitrap high-resolution mass spectrometry;
SDS-PAGE: Sodium dodecyl sulfate–polyacrylamide gel electrophoresis
