# Supplementary material for: Mining anticoagulant peptides from Poecilobdella manillensis by peptidomics analysis
Source: Nat Prod Bioprospect. 2026 Feb 2;16(1):24. doi: 10.1007/s13659-025-00573-0 (PMC12862037; doi:10.1007/s13659-025-00573-0)
Supplement: Supplementary file 1 — Additional file1 (DOCX 1163 kb) [file 13659_2025_573_MOESM1_ESM.docx]

**Mining Anticoagulant Peptides from *Poecilobdella manillensis* by Peptidomics Analysis**

Han-xue Zheng; Xiao-li Deng; Teng-teng Li; Guo-hua Xia; Huan Yang^*^; Jiang-song Peng^*^; Yu-ping Shen^*^

*Department of Chinese Materia Medica and Pharmacy, School of Pharmacy, Jiangsu University, Zhenjiang, Jiangsu, 212013, PR China*

^*^Corresponding author: Department of Chinese Materia Medica and Pharmacy, School of Pharmacy, Jiangsu University, Zhenjiang, Jiangsu, 212013, PR China. *E-mail* addresses: yanghuan1980@ujs.edu.cn (H. Yang), Jiangsong.Peng@outlook.com (J. Peng), syp131@ujs.edu.cn (Y. Shen)


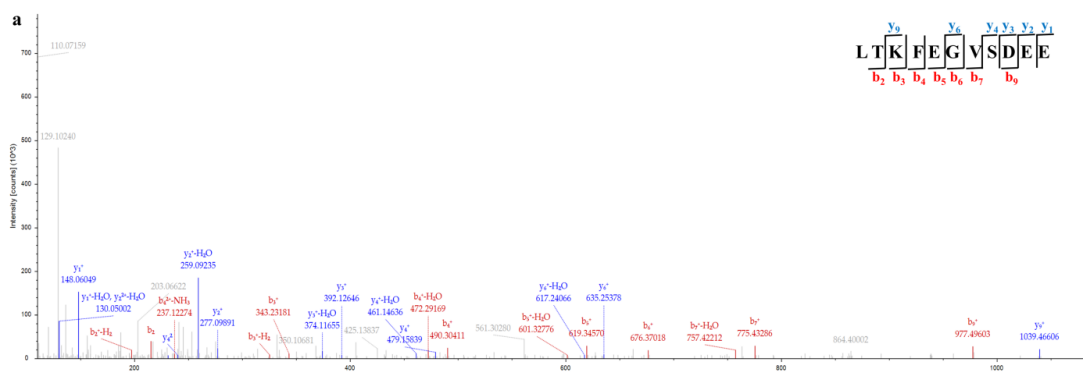


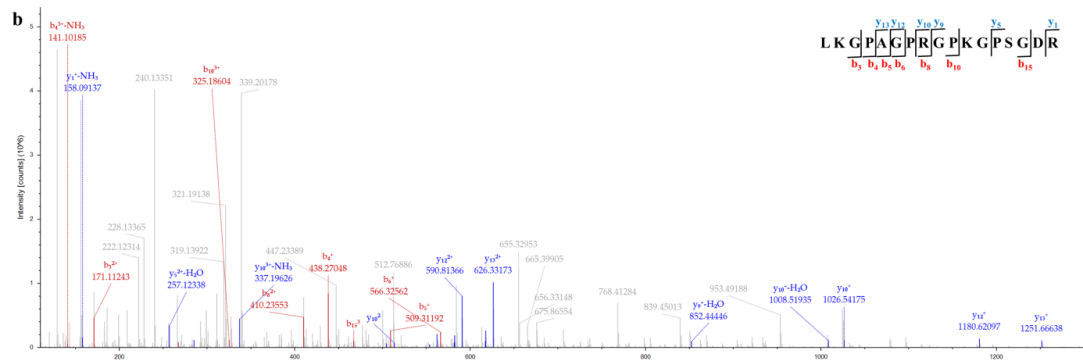


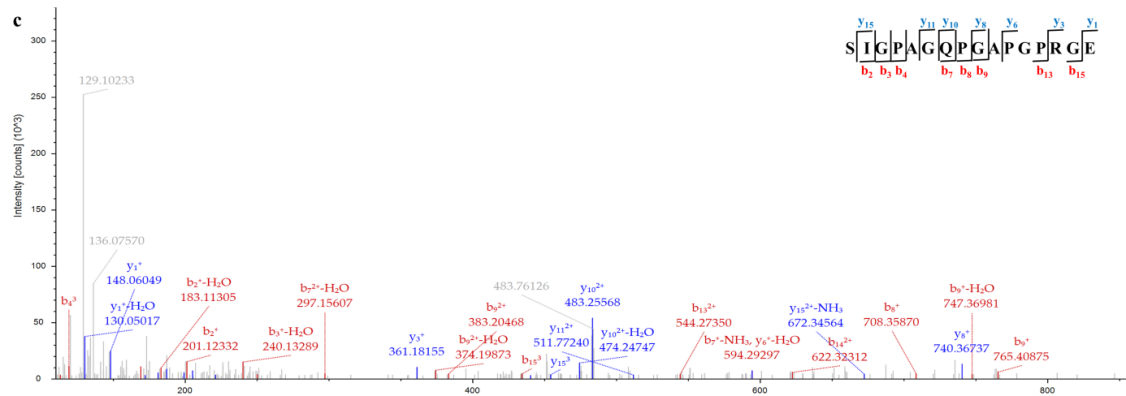


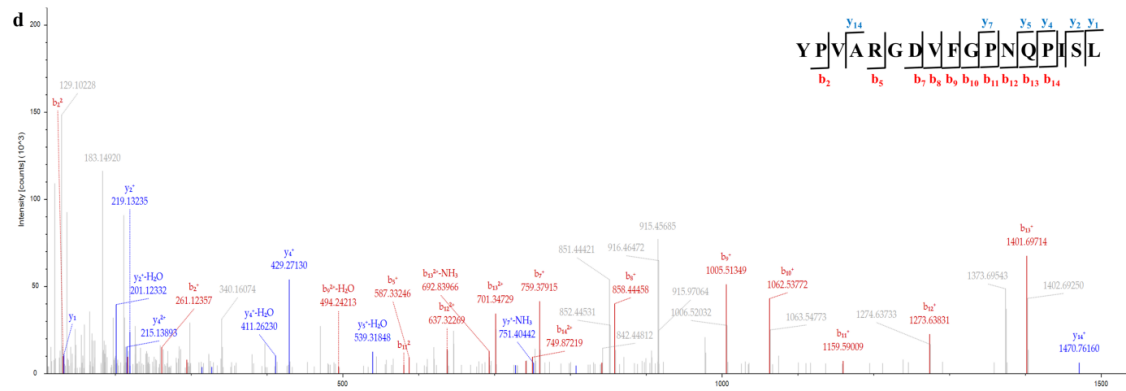


**Fig. S1** Secondary mass spectra of potential anticoagulant peptides

(a: LE-11, b: LR-17, c: SE-16, d: YL-17)

**
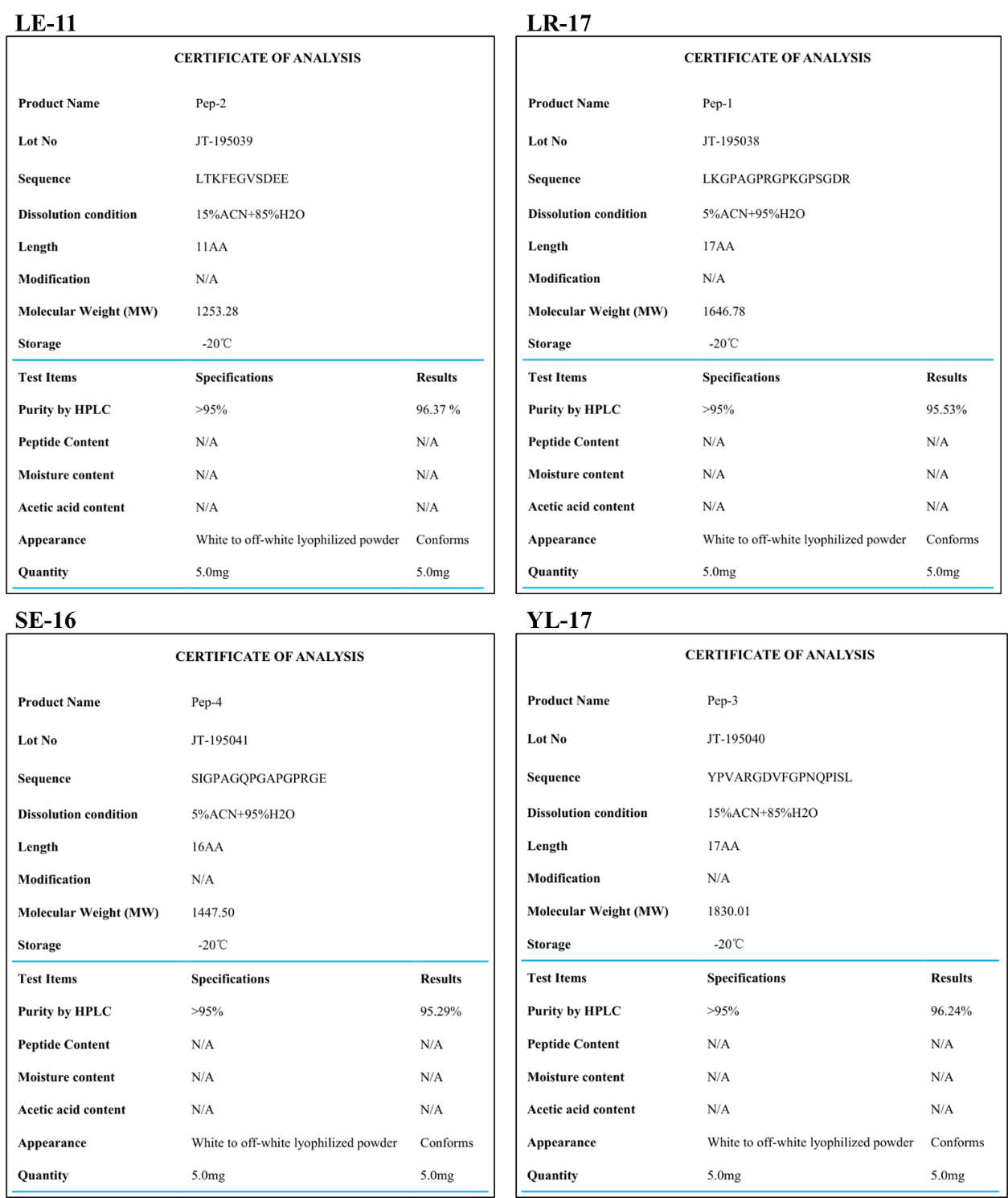
**

**Fig. S2** Certificates of analysis of four synthetic peptides


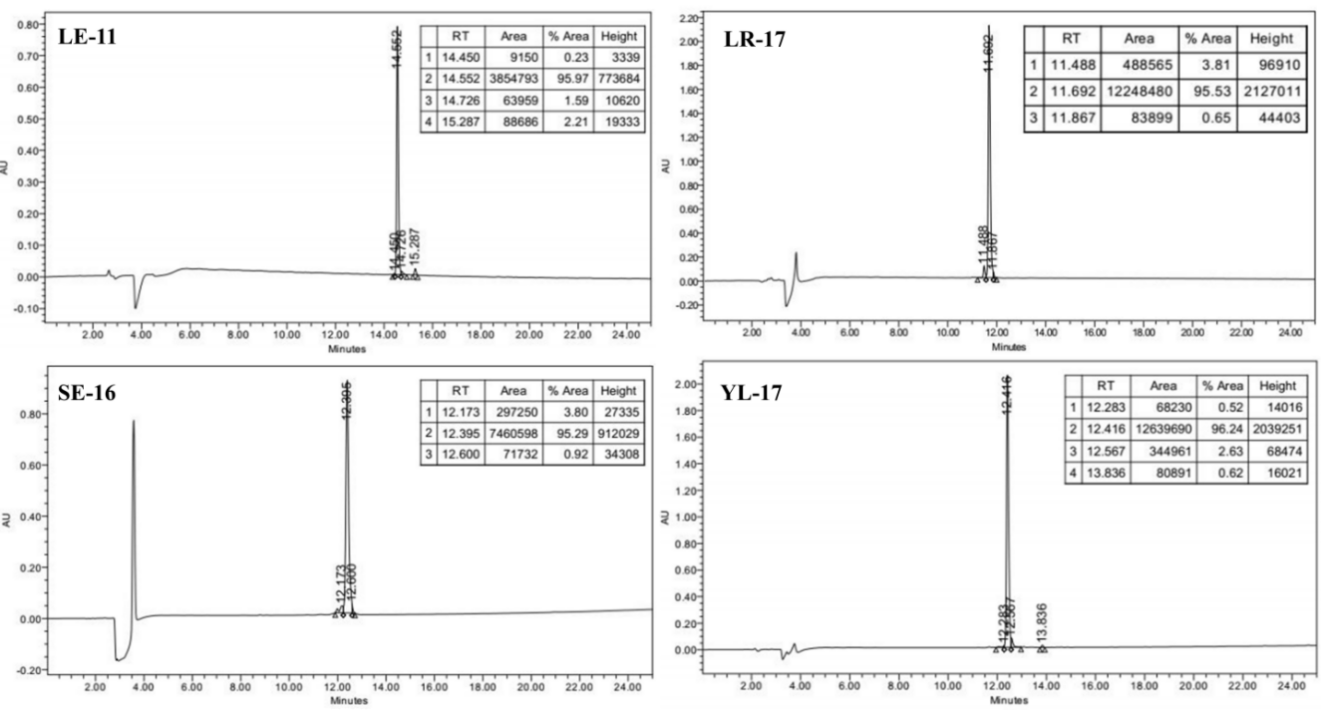


**Fig. S3** HPLC chromatograms of four synthetic anticoagulant peptides

**
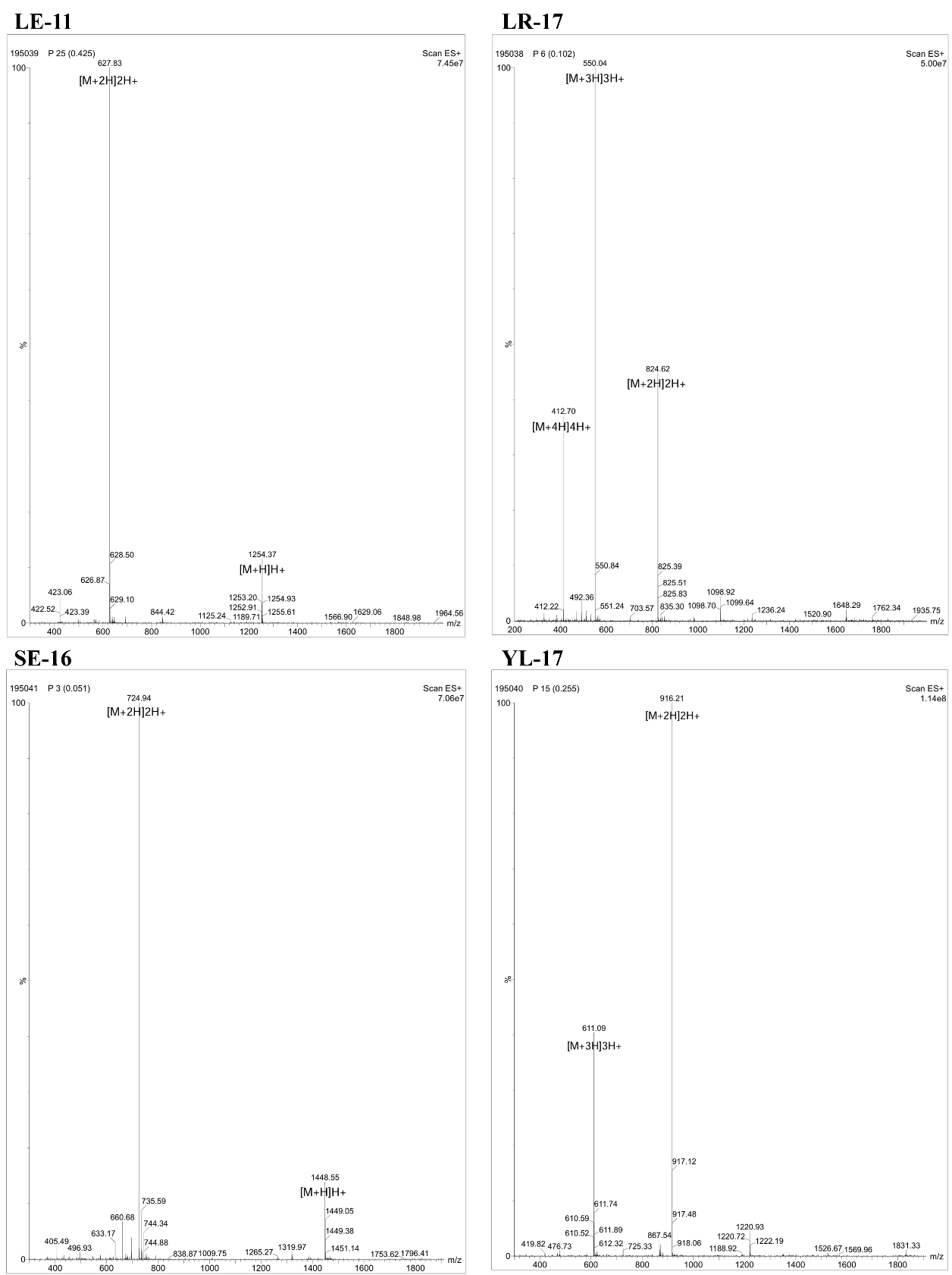
**

**Fig. S4** MS/MS spectra of four synthetic anticoagulant peptides


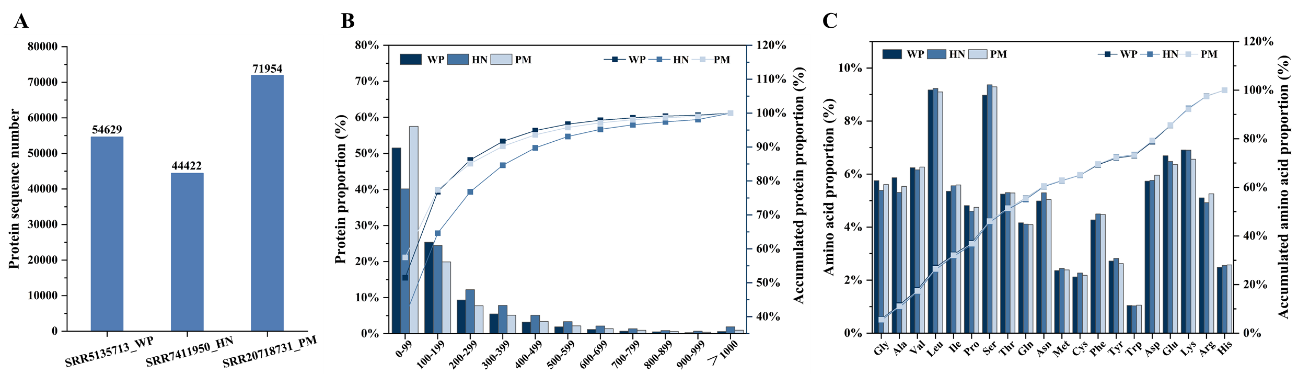


**Fig. S5** Analysis of the leech proteome database

A: Total protein sequence number; B: Distribution of protein lengths; C: Amino acid composition

**Table S1** Mass spectrometry parameters

|  | **Item** | **Value** |
| --- | --- | --- |
| Primary mass spectrometry | Resolution | 7000 |
|  | AGC targe | 3e6 |
|  | Maximum IT | 50 ms |
|  | Scan range | 350 to 3000 m/z |
| Secondary Mass Spectrometry | Resolution | 17500 |
|  | AGC target | 2e5 |
|  | Maximum IT | 45 ms |
|  | Loop count | 20 |
|  | TopN | 20 |
|  | Isolation window | 1.6 m/z |
|  | Fixed first mass | 110.0 m/z |
|  | (N) CE/stepped (N) CE | 28 |

**Table S2** Proteome Discoverer software data retrieval parameters

| **Item** | **Value** |
| --- | --- |
| Protein Database | hirudinea_0407; SRR5135713_WP; SRR7411950_HN; SRR20718731_PM |
| Enzyme | Enzymatic Unspecific |
| Max.Missed Cleavages sites | 2 |
| Precursor Mass Tolerance | 10 ppm |
| Fragment Mass Tolerance | 0.02 Da |
| Dynamic Modifications | Oxidation (M); Acetylation (N-term) |
| Static Modifications | No static modification |
| The false positive rate (FDR) | Less than 1% |

**Table S3** PRT measurement of *P. manillensis* proteins before and after biomimetic enzymatic digestion

| **Sample** | **PRT (s)** | **Intra-assay CV%** |
| --- | --- | --- |
| N | 40.67 ± 2.08 | 5.12% |
| PM-Pro | 269.67 ± 18.81**** | 6.68% |
| PM-Enz | 101.67 ± 13.87**** | 13.64% |

PRT data are presented as means ± SD (n=3). *****P*<0.0001 for the experimental groups versus the blank control (N)

**Table S4** PRT of three fractions PM-A, PM-B and PM-C

| **Sample** | **PRT (s)** | **Intra-assay CV%** |
| --- | --- | --- |
| N | 40.67 ± 2.08 | 5.12% |
| PM-A | 251.33 ± 5.51**** | 2.19% |
| PM-B | 191.67 ± 12.01**** | 6.27% |
| PM-C | 46.67 ± 0.58 | 1.24% |

PRT data are presented as means ± SD (n=3). *****P*<0.0001 for the experimental groups versus the blank control (N).

**Table S5** PRT of four subfractions PM-A1, PM-A2, PM-A3 and PM-A4

| **Sample** | **PRT (s)** | **Intra-assay CV%** |
| --- | --- | --- |
| N | 56.00 ± 1.00 | 1.79% |
| PM-A1 | 60.00 ± 3.61 | 6.01% |
| PM-A2 | 301.00 ± 9.54**** | 3.17% |
| PM-A3 | 67.00 ± 2.65* | 3.95% |
| PM-A4 | 45.67 ± 0.58* | 1.26% |

PRT data are presented as means ± SD (n=3). *****P*<0.0001 and **P*<0.05 for the experimental groups versus the blank control (N).

**Table S6** Assay of APTT, PT and TT of synthetic peptides

| **Sample** | **APTT (s)** | **Intra-assay CV%** | **PT (s)** | **Intra-assay CV%** | **TT (s)** | **Intra-assay CV%** |
| --- | --- | --- | --- | --- | --- | --- |
| N | 18.35 ± 0.77 | 4.20% | 13.67 ± 0.29 | 2.14% | 13.93 ± 0.38 | 2.73% |
| LR-17 | 17.17 ± 0.58 | 3.36% | 14.07 ± 0.55 | 3.94% | 16.23 ± 0.52*** | 3.23% |
| LE-11 | 32.14 ± 0.23**** | 0.72% | 13.66 ± 0.76 | 5.53% | 18.08 ± 0.28**** | 1.55% |
| YL-17 | 17.65 ± 0.26 | 1.50% | 13.72 ± 0.53 | 3.90% | 17.16 ± 0.51**** | 2.97% |
| SE-16 | 17.85 ± 0.71 | 3.98% | 12.59 ± 0.49 | 3.90% | 15.85 ± 0.50** | 3.16% |
| Hep-Na | 28.18 ± 0.23**** | 0.83% | 13.52 ± 1.03 | 7.58% | 23.01 ± 0.17**** | 0.73% |

Data are presented as means ± SD (n=3). ***P*<0.01, ****P*<0.001 and *****P*<0.0001 for the experimental and positive groups versus the blank control (N).
